# Priorities for research on environment, climate and health, a European perspective

**DOI:** 10.1186/s12940-022-00848-w

**Published:** 2022-03-28

**Authors:** Elina Drakvik, Manolis Kogevinas, Åke Bergman, Anais Devouge, Robert Barouki, on behalf of the HERA (Health and Environment Research Agenda) Consortium, Robert Barouki, Manolis Kogevinas, Åke Bergman, Elina Drakvik, Anaïs Devouge, Denis Sarigiannis, Delphine Destoumieux-Garzón, Franziska Matthies-Wiesler, Annette Peters, Daniel Zalko, Cristina Villanueva, Cathryn Tonne, Elisabeth Cardis, Elizabeth Diago-Navarro, Josep M. Antó, Maria Foraster, Mark Nieuwenhuijsen, Kurt Straif, Karin van Veldhoven, Kristine Belesova, Neil Pearce, Andy Haines, Jana Klánová, Kateřina Šebková, Lukáš Pokorný, Klára Hilscherová, Sandra Boekhold, Brigit Staatsen, Nina van der Vliet, Eeva Furman, Riikka Paloniemi, Aino Rekola, Marianne Aulake, Vivienne Byers, Alan Gilmer, Anke Huss, Roel Vermeulen, Rémy Slama, Michel Samson, Maria Albin, Åke Grönlund, Jeanne Garric

**Affiliations:** 1 Department of Environmental Science, Stockholm University, Stockholm, Sweden; 2 Department of Environmental Medicine, Karolinska Institutet, Stockholm, Sweden; 3 ISGlobal, Barcelona, Spain; 4 CIBER Epidemiologia Y Salud Pública (CIBERESP), Madrid, Spain; 5 Universitat Pompeu Fabra (UPF), Barcelona, Spain; 6 IMIM (Hospital del Mar Medical Research Institute), Barcelona, Spain; 7 Université de Paris, Inserm Unit, 1124 Paris, France

**Keywords:** Research agenda, Climate, Chemicals, Cities, Impact assessment, Infrastructures, Transformational change

## Abstract

Climate change, urbanisation, chemical pollution and disruption of ecosystems, including biodiversity loss, affect our health and wellbeing. Research is crucial to be able to respond to the current and future challenges that are often complex and interconnected by nature. The HERA Agenda, summarised in this commentary, identifies six thematic research goals in the environment, climate and health fields. These include research to 1) reduce the effects of climate change and biodiversity loss on health and environment, 2) promote healthy lives in cities and communities, 3) eliminate harmful chemical exposures, 4) improve health impact assessment and implementation research, 5) develop infrastructures, technologies and human resources and 6) promote research on transformational change towards sustainability. Numerous specific recommendations for research topics, i.e., specific research goals, are presented under each major research goal. Several methods were used to define the priorities, including web-based surveys targeting researchers and stakeholder groups as well as a series of online and face-to-face workshops, involving hundreds of researchers and other stakeholders. The results call for an unprecedented effort to support a better understanding of the causes, interlinkages and impacts of environmental stressors on health and the environment. This will require breakdown of silos within policies, research, actors as well as in our institutional arrangements in order to enable more holistic approaches and solutions to emerge. The HERA project has developed a unique and exciting opportunity in Europe to consensuate priorities in research and strengthen research that has direct societal impact.

## Background

Climate change, urbanisation, chemical pollution and disruption of ecosystems, including biodiversity loss, impact our health and quality of life. Research is instrumental to be able to respond to the current and future environmental and health challenges that are so complex and interlinked by nature. The European Commission (EC), in line with policies of the European Union and the United Nations Sustainable Development Goals [1], launched a call for proposals to define priorities for research on environment, climate and health [2]. The Health and Environment Research Agenda (HERA) project, emerging from that call, was developed by a European consortium, and recently submitted its final report entitled “EU research agenda for the environment, climate & health, 2021–2030” [3], summarised in this commentary. The HERA Agenda highlights several key areas where further research is crucial for the next decade. This article provides a topical contribution to discussion of environmental health priorities and provides opportunities to reflect on future directions of research in this field, especially in the European context.

## Process for developing the European research agenda

**Fig. 1:**
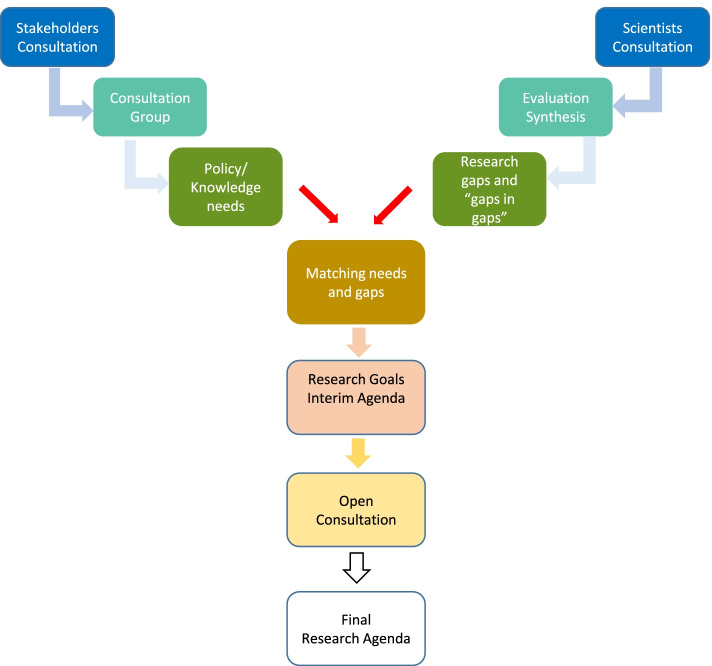
HERA framework for engaging stakeholders and scientist in the definition of Research goals (RG) for the research agenda

The approach that the project followed was based on principles of transparency, inclusiveness and mutual learning [4]. During the course of the project, the HERA Consortium performed extensive reviews of current knowledge, policies and research activities (Fig. 1). Web-based surveys targeting research communities and other stakeholder groups were carried out, along with online and face-to-face workshops, which taken together, involved hundreds of participants. Researchers primarily identified major current areas of concern, i.e. air pollution, chemicals, climate, cities, as priorities for research. Other stakeholders mostly identified implementation science and global issues (e.g. climate change, biodiversity loss) as priorities. The stakeholder process and results are described in detail in Paloniemi et al. [5]. Responses from the surveys and workshops were discussed by the HERA working group that further identified “Gaps in gaps”, namely research areas that are not well developed but that were not identified by the researchers’ survey. Research goals were prioritised by the HERA working group using the following criteria (modified from [6]): Novelty; Importance to People, Importance to the environment on a planetary scale; Impact on Policy; and, Innovation and Sustainable Development. A consensus-based approach was used for agreeing and refining the research goals along the process, based on the input and expertise of the HERA Consortium members, editorial group and independent scientific advisory board as well as input received through a public consultation.

## The EU research agenda for the environment, climate & health, 2021–2030

The EU Research Agenda developed by the HERA project covers six major research goals on environment, climate and health. Within each of them, research areas were identified and research needs specified resulting in altogether 30 specific research goals (Table 1). Several of the research goals are interlinked e.g. air-pollution is identified as a priority in the global environment (Research Goal 1.6 Global pollution) and the local environment, cities and communities (Research Goal 2.2 Air pollutants in indoor and outdoor environments). Moreover, the Research Agenda addresses research that can contribute to relevant policy objectives promoting health and the environment, especially in the context of the European Green Deal [7]. The Green Deal aims at achieving climate neutrality, biodiversity preservation, a circular economy and a zero pollution/toxic-free ambition as well as providing a way forward for achieving sustainable food system. The HERA agenda and the identified research needs can hence strengthen the knowledge and evidence-base in these cross-cutting policy areas, directly supporting the implementation of the Green Deal.

**Table 1:** Priorities in environment, climate and health research, HERA project. Six Research Goals and 30 specific Priorities

Research goal 1 “Climate change and biodiversity loss – reduce effects on health and the environment”
RG1.1 Health and climate change
RG1.2 Health impacts of climate mitigation and adaptation measures
RG1.3 Health and biodiversity loss
RG1.4 Biological agents, environment and human health
RG1.5 Food, ecosystem services and farming
RG1.6 Global pollution
Research goal 2 “Cities and communities – promote healthy lives in sustainable and inclusive societies”
RG2.1 Healthy Urban Environments
RG2.2 Air pollutants in indoor and outdoor environments
RG2.3 Noise in living environments
RG2.4 Changing work and employment conditions
RG 2.5 Digitalisation, changed mobility patterns and effects on environment and health
RG2.6 Waste and contaminated sites
Research goal 3 “Chemicals and physical stressors – prevent and eliminate harmful chemical exposures to health”
RG3.1 Exposure to chemicals including legacy chemicals, emerging chemicals and mixtures
RG3.2 Health effects of anthropogenic chemicals
RG3.3 Radiation
RG3.4 Water contamination
RG3.5 Food and soil contamination
Research goal 4 “Improve health impact assessment of environmental factors and promote implementation research”
RG 4.1 A unified European approach on quality of life and burden of disease
RG 4.2 Develop tools and methodologies for integrative environmental health risk assessment
RG 4.3 Advance and systematise implementation research in environment and health
Research goal 5 “Develop infrastructures, technologies and human resources for sustainable research on environment, climate change and health”
RG5.1 Well-designed and maintained population cohorts and related biobanks
RG5.2 Development of laboratory capacities for assessment of the chemical exposome and its functional impacts
RG5.3 Innovative big data-based methods and tools to characterize interrelationships between environment and health
RG5.4 Transdisciplinary research infrastructure: Planetary Health monitoring
Research goal 6 “Promote research on transformational change in environment, climate change and health”
RG6.1 Preparedness to prevent and combat future environment and health threats/challenges
RG6.2 Transformational change
RG6.3 Socioeconomic factors and the environment, environmental injustice, equity, sustainable economic growth
RG6.4 Ethical, philosophical and political aspects
RG6.5 Science communication and science–policy-society dialogue
RG6.6 Transformational change in education, training and research

### The six overarching Research Goals

- **Research goal 1 “Climate change and biodiversity loss – reduce effects on health and the environment” **focuses on global interconnected issues. The consequences of climate change, biodiversity loss, disruption of food chains, emerging infectious diseases and decreased ecosystem services on health are not well understood despite evidence that they have major and persistent effects on life and the environment globally that became evident from the COVID-19 pandemic. Furthermore, more attention is required for addressing pollution, including air pollution, at a global scale. The need to promote research for effective policies on mitigation and adaptation is identified as of paramount importance, as well as investigating co-benefits with air pollution mitigation policies. Overall, the research goal highlights the need for holistic approaches such as One Health and Planetary Health.
- ***Research goal 2 “Cities and communities – promote healthy lives in sustainable and inclusive societies” ***focuses on problem-based research. Living conditions in urban environments are of key concern as they impact the health and wellbeing of most European citizens. The impacts of environmental factors (e.g. air pollution, noise, digitalisation), may vary in different contexts such as the urban environment workplace or contaminated land. Research should examine the complex relationships in these environments, and evaluate and promote positive interventions.
- ***Research goal 3 “***Chemicals*** and physical stressors – prevent and eliminate harmful chemical exposures to health”*** focuses on chemicals, other stressors and environmental media. There are still many unknowns on the hazards and risks related to stressor families including chemicals and mixtures, physical stressors such as radiation (ranging from ionising to light exposure), and the role played by the various environmental media carrying these stressors such as water. Research should cover the tens of thousands of chemicals in daily usage that we have very little health information on and interactions of environmental exposures with other factors such as genes, occupation, political and socioeconomic determinants of health, a theme covered also in RG6 on interdisciplinary research. Regulatory decisions rely heavily on additional knowledge in these specific areas. Research should effectively address the challenges of a zero pollution paradigm and a sustainable future.
- ***Research goal 4 “Improve health impact assessment of environmental factors and promote implementation research”*** focuses on the need to develop new harmonized methodologies to evaluate the burden of environmental and climate change on health and to identify and assess the health benefits of human environmental interaction. Moreover, research should promote optimal ways to implement science-based decisions and policies as this is a limiting factor in many fields.
- **Research goal 5 “Develop infrastructures, technologies and human resources for sustainable research on environment, climate change and health”** focuses on the need of European research infrastructures to be strengthened and further developed. Infrastructures provide a basis for excellent research. Key proposals are establishing harmonized coordination of ongoing large cohort studies including tens of millions of participants, exposome characterization, laboratory infrastructure, data analysis using the latest data science tools, new methods for exposure assessment (e.g. sensors) and planetary monitoring tools.
- **Research goal 6 “Promote research on transformational change in environment, climate change and health”** focuses on the need of transformational change to address the intertwined environmental, social and health issues and reach critical global goals towards sustainability and equity. Societies will need to adapt to the challenges elicited by environmental stressors and climate change and this will require significant transformation of individual and collective behaviour and of policy making across the sectors and silos. Development of research approaches directed to finding and promoting workable solutions together is necessary for achieving such transformations.

## Conclusions—a vision for future research

It is striking how the HERA surveys and stakeholder consultations pointed out such a large number of knowledge gaps, even in areas such as climate change where relevant evidence-based policies are urgently needed. The ambitious political goals set in the UN Agenda for Sustainable Development and the European Green Deal, will need major investments in research and innovation. The HERA Agenda coincides with the reports highlighting the planetary boundaries [8, 9], and intertwined environmental pressures, the triple planetary crisis: climate change, biodiversity loss and pollution, affecting the health of the planet and of the people [10]. The Agenda reinforces the opportunity to bring together human health and environment field to work together on integrated and transformative solutions. The focus is on Europe, hence putting less emphasis on major exposures, such as indoor air pollution from biomass, that are much more prevalent in low- and middle-income countries. In fact, there is an urgent need to also develop a global Agenda since most of the problems and solutions discussed in HERA are not limited to Europe. In recent years, increases in the EU allocation to environment and health projects through the Framework Programme budgets and rise in the interest and importance of the field ([11], see page 65), have not yet managed to close the long-term gap that exists between required research and funding. It is a positive signal that the HERA Agenda has already been applied by the European Commission in recent calls for funding, as for example calls for the indoor environment, or planned calls on planetary health or the interlink of infections and the environment. Nevertheless, the vision for future research underlying this Agenda calls for an unprecedented effort to support a better understanding of the causes, interlinkages and impacts of environmental determinants on health. Integrated and holistic research should support policies and practices to protect and promote human health and well-being while simultaneously improving the critical state of the environment, including climate change mitigation and ecosystem restoration, in Europe and globally. This requires transformational change at societal level to break down the silos in policymaking, research, and institutional arrangements, enabling cross-sectoral, interdisciplinary and holistic approaches and solutions to emerge.

## Data Availability

Not applicable.
